# Evaluation of Water Scarcity Footprint for Taiwanese Dairy Farming

**DOI:** 10.3390/ani9110956

**Published:** 2019-11-12

**Authors:** Wei-Tse Liao, Jung-Jeng Su

**Affiliations:** 1 Department of Animal Science and Technology, National Taiwan University, Taipei 10673, Taiwan; r06626023@ntu.edu.tw; 2 Department of Animal Industry, Council of Agriculture, Executive Yuan, Taipei 10014, Taiwan; 3 Bioenergy Research Center, College of Bio-resources and Agriculture, National Taiwan University, Taipei 10617, Taiwan

**Keywords:** life cycle assessment, water scarcity footprint, water scarcity productivity, raw milk, sustainability

## Abstract

Raw milk production in Taiwan has increased year after year, which means that the environmental impact might also be intensified in certain regions. To balance both consumer demand and environmental sustainability, evaluating the potential impact and understanding the causal relationship between production and environment is imperative. This study applied the life cycle assessment (LCA) protocol to explore water consumption for raw milk production from cradle to farm gate of five dairy farms in Hsinchu County and evaluate the stress-weighted water scarcity footprint (WSF) as well as the water scarcity productivity (WSP) of the 16 Taiwanese counties and cities. Results indicated that the highest stress-weighted WSF of the dairy farms for raw milk production was located in northern and central Taiwan and was around 44.8 H_2_O_eq_/kg fat- and protein-corrected milk (FPCM). On the other hand, both the smallest stress-weighted WSF (about 2.2 H_2_O_eq_/kg FPCM) and the highest WSP (0.749 kg FPCM/m^3^ water) of the dairy farms were located in Nantou and Chiayi Counties, because these two counties were the least water-stressed regions in Taiwan. The achievement of this study could be the first and important reference for the sustainable production of raw milk and optimizing the industrial policy of dairy farming by policy makers.

## 1. Introduction

With the increase of national income and living standards in Taiwan, increased demand for dairy products has stimulated rapid growth of the dairy industry. In 1980, Taiwan’s dairy farms raised 23,636 cows with an annual milk production of 50,154 t [1]. In 2017, the number of dairy cows increased to 130,413 heads, while the annual milk production equaled 386,362 t [2]. The continued expansion of the farm scale means more water is needed for operation, but it should be noted that water is a very limited resource in Taiwan. 

The most common water resource for most Taiwanese dairy farms, as well as other livestock farms, is groundwater. In the past time, groundwater well digging was not forcibly regulated by agricultural laws. Thus, management of the existing groundwater wells and control of new well digging will be a tough task for releasing water scarcity stress in some regions. In 2013, the Council of Agriculture (COA) of Taiwan proposed a five-year “Golden Gallery New Agriculture Action Plan” to control the conditions of ground subsidence along with areas of the high-speed railroad (Available online: https://book.tndais.gov.tw/Magazine/mag87/87-1.pdf; accessed on 1 November 2019). The action plan aims to reduce the number of existing groundwater wells and subsidize wastewater recycling systems of livestock wastewater treatment in the livestock farms located in the regions of Yunlin County and Changhua County.

Water footprint assessments based on ISO 14046 are primarily focused on water and related environmental impacts. Water footprint inventory analysis (i.e., life cycle inventory, LCI) involves compilation and quantification of inputs and outputs related to water in various production processes in life cycle stages. Inventory data were collected to describe the cow breeding and dairy processing. Only the consumptive water extracted from surface and groundwater were considered. Following the water footprint inventory analysis, a water footprint impact assessment (i.e., life cycle impact assessment, LCIA) was used to evaluate the magnitude and significance of the potential environmental impacts due to changes in water quantity and quality [3]. 

In a particular region and period, if the rate of water consumption exceeds water supplement, it will be a form of resource depletion, which limits water availability for future users [4]. Improper water management will endanger biodiversity, disrupt ecological functions, disturb hydrology, and even change the climate [5]. Water scarcity footprint (WSF) is used to evaluate potential environmental impacts related to water scarcity resulting from an enterprise’s activities in an area [6]. The water stress index (WSI), which was based on a withdrawal-to-availability ratio according to the method proposed by Pfister [7], was used as a local characteristic factor, e.g., watershed, region, nation, etc. Effective identification of water hotspots and major impacts in raw milk production have been studied [6,8,9,10,11,12,13,14]. However, in these cases, due to data limitations or partial water use of projects that were too large, the results might lead to inappropriate improvements [15]. 

**Figure 1:**
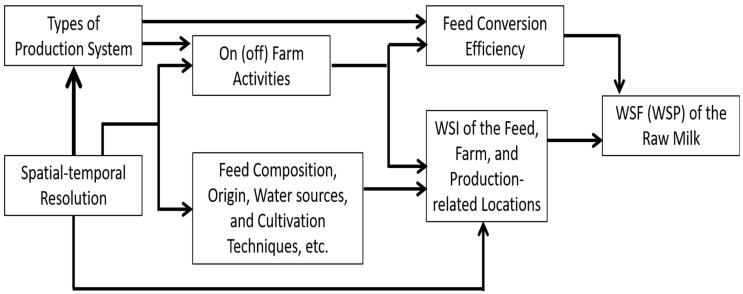
Factors that might affect the water scarcity footprint (WSF) or water scarcity productivity (WSP) of raw milk.

There are many factors affecting the analytic results of WSF in raw milk production (Figure 1). The measurement must be performed with caution and the results should be explained correctly. Otherwise, it might create a misunderstood among the public, for example, assuming that the lower the value the better, but people might skip other factors important to production. Dairy farmers may be contradictory about the requirement of low WSF and water consumption for high raw milk production in terms of water productivity. Water productivity (WP) is defined as the physical or economic product output per unit of water application [16]. If they combined the WSI concept with the water scarcity productivity (WSP), the public and dairy farmers could easily understand the meaning and direction of improving dairy production.

Because of rapid industrial development leading to high water consumption (e.g., integrated circuit foundry) in Hsinchu County, in addition to the effects of climate change, the reasonable and fair distribution of water resources for industries and agriculture is the most disputed issue during the dry seasons annually. The strategy for sustainable utilization of water resources has been studied by the local government, which included strengthening land use management, improving agricultural resilience, and implementing water footprint (WF) management [17]. Therefore, the objectives of this study are to analyze the water use on raw milk production in Hsinchu County and evaluate the WSI, WSF, and WSP of dairy farming in Taiwan. In addition, this study attempts to summarize a concrete, feasible, and sustainable strategy as a reference for policy makers and the related dairy industry.

## 2. Materials and Methods

### 2.1. Selected Dairy Farms

Five commercial dairy farms were selected for this study based on the use of local water resources (i.e., groundwater or surface water), the willingness of participation, and routine operation for more than one year. All selected dairy farms used sawdust or rice husk as bedding. The total number of dairy cattle on farm for the Farm#1, 2, 3, 4, and 5 was 203, 374, 211, 395, and 369, respectively (Table 1). Thus, the average number of cattle on the selected five dairy farms was about 310 ± 95 heads and the workload was about 53 ± 11 head/labor individually. The daily raw milk production of these five dairy farms was 29.3, 27.6, 24.2, 23.5, and 28.9 kg, respectively (Table 1). The parameter of the water trough supply was 16.9, 31.2, 26.5, 43.9, and 21.7 heads/water trough, respectively (Table 2). The number of dairy cows in Hsinchu County was 2050, accounting for 2% of the country’s total size [2]. The total dairy cattle of the farms within the housed system are 1498 heads, accounting for about 83% of the Hsinchu County’s total dairy cattle number on farms (1807 heads as of May 2018). 

### 2.2. System Boundaries

**Figure 2:**
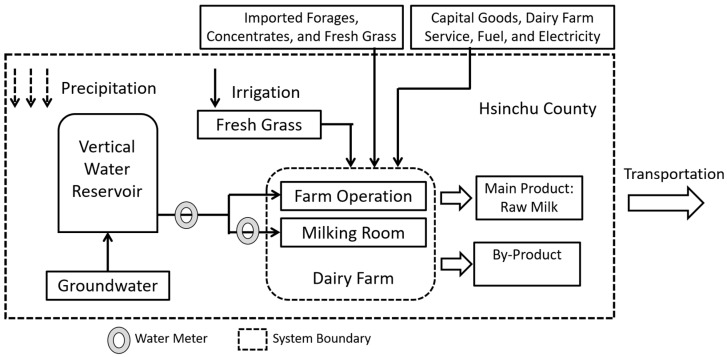
System boundary of the raw milk production in Hsinchu County.

The Hsinchu County is located in the north of Taiwan and the average annual precipitation is 1742 mm [17], which is a region that lacks water resources. The system boundary was defined from cradle (feed crop cultivation) to farm gate (Figure 2). Dairy cow diets were mainly composed of roughage (i.e., fresh forage and imported forage) and concentrates. Imported forages and concentrates are excluded from system boundaries due to the lack of life cycle data, and it is not feasible to change the country based on factors other than the quality and price of the imported forage. Moreover, the raw milk production cost in Taiwan is about USD 0.42–0.6/kg, which is greater than the cost in U.S. (USD 0.2/kg) and New Zealand (USD 0.11/kg) [1]. The inventory at cradle stage was limited to the fresh forage cultivated at Hsinchu County (forages purchased from other counties and cities are not in line with the purpose of this study). 

### 2.3. Data Collection

Following the ISO 14046 guidelines, the data of inventory production and water consumption of these five dairy farms were collected monthly by installing water meters, and the inventory timeline is one year, from May 2018 to April 2019. The details of water consumption for each dairy farm were found by estimating the in situ survey data individually. The inventory production included feed crops production, raw milk production, raw milk fat, raw milk protein, and herd composition. Water consumption included water for irrigation and operation of farm, such as drinking water, cleaning water, mixed water of total mixed ration (TMR), cooling water, etc. 

Twelve velocity water meters (IH-13, I-Hao Essence Mechine Co., Taiwan) were installed in the well and the milking room to monitor water consumption (Figure 2), the specifications of which are shown in Table 1. The remaining water consumption is estimated according to the operating pattern. The consumption of soil moisture derived from natural precipitation does not generally contribute to regional water scarcity in water bodies; therefore, it was not included in the calculation. Capital goods used in production (e.g., machinery and buildings), domestic water, and dairy farm services (e.g., veterinary and business services) were not included in the assessment because the environmental relevance of these items is difficult to ascertain and minor for most dairy farming systems [9,10,11,13,14,18]. In order to compare with other studies, the chosen functional unit is 1 L of fat and protein corrected milk (FPCM; 4.0% fat and 3.3% protein) [19]. An economic approach was used to allocate water consumption between raw milk and by-products (e.g., cow dung, culled dairy cattle, calves, etc.), the economic value of which was based on the average prices during the period of 2013–2017 [20,21].

### 2.4. Impact Assessment

This study used the methodology of Ridoutt and Pfister [4] to evaluate the WSF from raw milk production in Hsinchu County. Calculation of the freshwater resources consumed per unit of FPCM at each stage of production was based on the data from the inventory and the economic allocation method. The values of volume from the previous calculation were then multiplied by the local WSI to obtain the WSF value. The WSI index value ranged from 0 to 1, which serves as a characterization factor for a suggested midpoint category “water deprivation” in LCIA. The WSI indicates the portion of consumptive water use that deprives other users’ fresh water. The severity of water scarcity of water sheds is ranked as follows: WSI < 0.1 (low); 0.1 ≤ WSI < 0.5 (moderate); 0.5 ≤ WSI ≤ 0.9 (severe), and WSI > 0.9 (extreme) [7,10,22]. The available precipitation in each county and city are taken from the “CWB (Central Weather Bureau, Taiwan) Observation Data Inquire System (CODiS)” from 2009 to 2018 (https://e-service.cwb.gov.tw/HistoryDataQuery/index.jsp). Moreover, the second discussion meeting of “strengthening water resources allocation measures and strategy for agricultural water saving” was held and hosted by the COA [23,24]. Thus, the domestic data for this study were based on the water use data of the meeting, including agricultural water, industrial water, and domestic water from 2004 to 2015. The stress-weighed WSF results might be related to an equivalent volume of freshwater consumption at the global average WSI [4,10]. 

### 2.5. Water Scarcity Productivity (WSP)

Water productivity (WP) is defined as the physical quantity or economic value produced by a unit of water [16]. In this study, we combine WP with the WSI as the equation:

$$\mathrm{WSP}\left(\mathrm{kg}/m^{3}\right)=\frac{P_{i}\left(\mathrm{kg}\right)}{{\mathrm{WC}}_{i}\left(m^{3}\right){\mathrm{WSI}}_{i}}$$

Here the WSP (kg/m^3^) is for water scarcity productivity; P*_i_* (kg) is for FPCM production per unit time in position *i*; WC*_i_* (m^3^) is for water consumption per unit time in position *i*; WSI*_i_* is for characteristic factors corresponding to P*_i_*; *i* is for different geographical location (e.g., watershed, region, nation, etc.). WSP can be increased by either increasing FPCM yield or reducing WC and maintaining the yield level or changing the production location to a low WSI region.

## 3. Results and Discussion

### 3.1. Water Consumption by In Situ Measurement and Estimation

The water consumption of the five dairy farms varied according to their management style and the number of dairy cattle on the farm. The results of in situ water meter measurement showed that the monthly water consumption was 962, 1613, 348, 1401, and 1298 m^3^, respectively, from May 2018 to April 2019 (Table 3). The amount of water consumption was proportionate to the number of dairy cattle on the farms. However, the estimated amount of water consumption on Farm#1 was about three times the water consumption on Farm#3. These farms had almost the same number of dairy cattle on farm. The results showed that Farm#1 spent more water on cleaning water troughs (18%) and milking (15%), but Farm#3 spent more water only on milking (24%) except for drinking purposes (Table 3).

The estimated data by individual in situ survey showed that there about 50 to 65% of water consumption was for animal drinking and 2 to 19% of water consumption for cooling the animal house. The average water consumption of the five dairy farms for the milking rooms, cleaning troughs, cooling animal houses, TMR mixing, cleaning the farms, miscellaneous, and drinking was about 9.8, 6.8, 13.4, 6.1, 6.8, 0.2, and 52%, respectively. The results showed that Farm#2 spent more water on cooling the animal house (19%) and cleaning (11%), but Farm#5 spent more water on cooling the animal house (18%) and milking (12%) except for drinking purposes (Table 3). Only Farm#4 spent more water on TMR mixing (11%), cleaning (11%), and milking (11%) except for drinking purposes (Table 3). In summary, different management styles can result in different water consumption situations on dairy farms. Water consumption for cleaning was about 1–11% of the total water consumption for all selected dairy farms, because the selected dairy farms applied sawdust or rice husk as bedding (Table 3). The frequency of water-consuming categories, i.e., milking, cleaning trough, cooling, TMR mixing, cleaning, miscellaneous work, and drinking, mainly affects the estimated water consumption data based on in situ farmer surveys (Table 3). 

**Figure 3:**
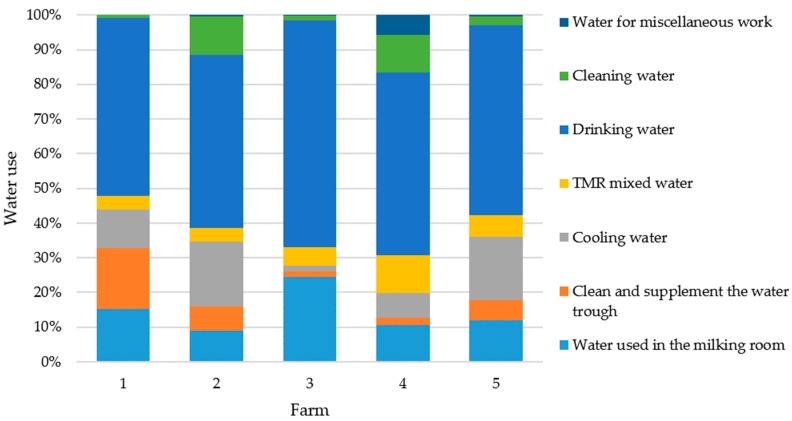
The average proportion of water consumption in individual dairy farm.

The local FPCM annual output was 2333 t and the average daily FPCM yield is 9.2 kg. The Farm#4’s daily FPCM yield was the lowest (8.0 kg), but Farm#1’s was the highest (10.1 kg). Results showed that the average water consumption per kilogram of FPCM was 27 ± 7 L. Farm#1 was the highest user of water resources (up to 34.9 L) and Farm#3 was the lowest user (17 L). The five dairy farms consumed a total of 67,464 m^3^ of water. Both Chinese Pennisetum (200 t) and Pangola grass (479 t) were harvested as part of the feed components. The water sources used to grow these forages were either from rainfall or the effluent from a dairy cattle wastewater treatment facility, which were not included in the water consumption calculation. The average of water use distribution for all dairy farms showed that drinking water was the highest proportion (up to 55%) and the percentages of water used in the milking room and cooling water were 14 and 12%, respectively (Table 3). The water use ratios for each dairy farm are shown in Figure 3.

### 3.2. Water Stress Index (WSI), Stress-Weighted Water Scarcity Footprint (WSF), and Water Scarcity Productivity (WSP)

**Figure 4:**
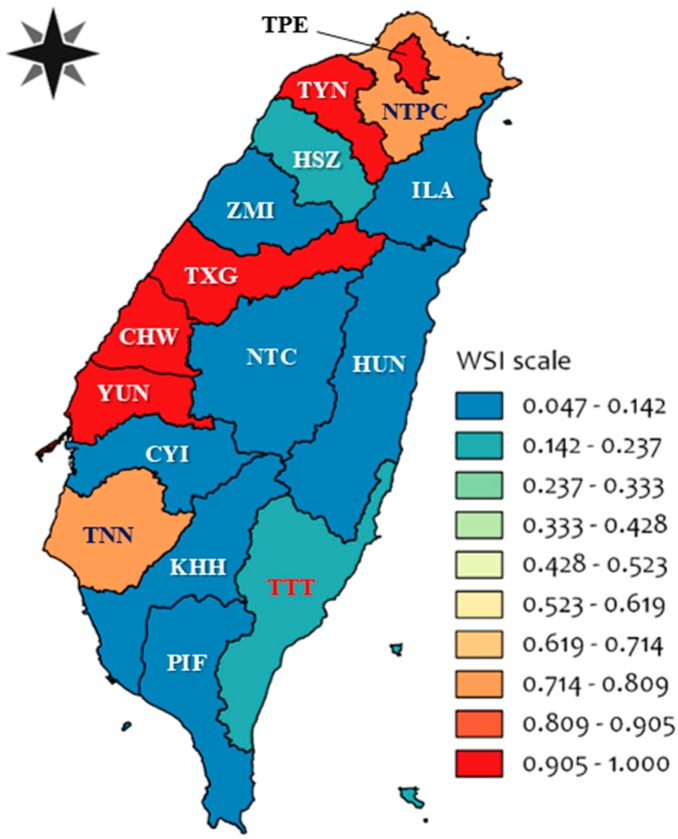
The water stress index (WSI) in various regions of Taiwan (made by QGIS 2.8).

The WSI, stress-weighted WSF, and WSP data of various counties and cities in Taiwan are shown in Table 4. The counties and cities with higher stress-weighted WSF were mostly located in northern and central Taiwan (Figure 4) including Taipei City (44.8 H_2_O-eq/kg FPCM), Taoyuan City (44.8 H_2_O-eq/kg FPCM), Taichung City (44.8 H_2_O-eq/kg FPCM), Changhua County (44.8 H_2_O-eq/kg FPCM), Yunlin County (44.3 H_2_O-eq/kg FPCM), Tainan City (34.5 H_2_O-eq/kg FPCM), and New Taipei City (32.7 H_2_O-eq/kg FPCM). The WSI of those counties and cities all reached 0.99 and the severity of water scarcity was under the “extreme” condition. The data of stress-weighted WSF in southern (except Tainan) and eastern Taiwan were low, from 2.2 to 6.7 H_2_O-eq/kg FPCM. The data of WSP for all counties and cities also pointed out that the counties with low WSI, such as Nantou and Chiayi County (WSI = 0.05), had the highest WSP of 0.749 kg/m^3^. However, the lowest WSP was 0.037 kg/m^3^ in Taipei City, Taoyuan City, Taichung City, and Changhua County, which were under “stress” conditions because of the local water resources. 

### 3.3. Raw Milk Yield, Quality, and Pricing

For the milk processing industry, if demand of the environment-friendly product manufacturing increased, the comparison of raw milk sources by WSF evaluation process might be more important. The results of WSP showed that the same production method might be more productive where the water stress was relatively lower. Water scarcity productivity (WSP) combines regional water stress with the physical quantity or economic value produced by a unit of water, which might be easier and more intuitive for farmers to improve their production efficiency. The explanation for general consumers may also be simpler than WSF. The results of WSF might provide the thought that the lower amount of water consumption, the better for raw milk production. 

Lactating cows require a larger portion of water relative to their body weight (BW), since 87–88% of milk is water. Water intake requirements are influenced by physiological state, rate of milk yield, dry matter intake (DMI), body weight, composition of diet, and environmental factors [25]. Increasing air temperature, temperature–humidity index (THI) and rising rectal temperature above critical thresholds are related to decreased DMI and to reduced efficiency of milk yield as well as milk quality [26]. Water is arguably the most important nutrient for the dairy cow. Water intake is closely related to DMI and milk yield, but minimum temperature was the second variable to enter a stepwise regression equation (after DMI) [27]. Water intake increased by 1.2 kg/°C in minimum ambient temperature, but regardless of the rate of increase, it is clear that abundant water must be available at all times under hot conditions. In addition, it has been demonstrated that offering chilled drinking water enhances milk yield for lactating cows [28] by reducing body temperature through absorbed heat energy. Moreover, dairy cows need water for growth, digestion, metabolism, pregnancy, lactation, milking, and cleaning of milking equipment and barns [29]. Sufficient water supply can help all dairy farms to take good care of the cows, produce good raw milk, and gain profit. 

Because about 87–88% of milk is water, sufficient water supply and DMI may help dairy farms to produce good raw milk and gain more benefits. The price of Taiwanese raw milk is mainly based on the contents of both solids-non-fat (SNF) and raw milk fat in milk [30]. The raw milk pricing table (USD 0.54–1.05/kg-raw milk) is based on the SNF content (8.00–9.18%), raw milk fat (2.8–4.0%), and seasonality (Table 5). The milk consumption season is mostly in summer, thus, the raw milk price in warm seasons (i.e., summer) is higher than temperate and cool seasons, in consequence. 

Feeding 1 kg of dry matter and lactating 1 kg of raw milk requires 4–5 L and 3–5 L of fresh water for each lactating cow after milking, respectively [31]. Milking 16 to 20 L of raw milk requires 70 L of fresh water per lactating cow daily, but a dry cow only requires 40 L of fresh water daily. The minimum fresh water supply time is about 4 times a day for maintaining optimal milk production. In summary, sufficient water supply for drinking, feeding, and cooling is very important to ensure raw milk quality, milk production efficiency, and animal health.

### 3.4. Fresh Water Supply for Efficient Raw Milk Production

The specification and parameters of the water troughs on dairy farms are shown in Table 2. The parameters showed that the dairy cattle of the Farm#1 and #5 owned the best allocation space of water trough supply. The lengths of the allocated drinking water trough are only 3.7 and 5.9 cm for cows raised in Farm#3 and #4, respectively (Table 2), which is lower than the recommended standard of 6.4 cm. In addition, the water trough set in Farm#2, #3, and #4 was also higher than the recommended standard of 25 cows [31]. These conditions might impair the access of drinking water for dairy cows, which may lead to reduced water consumption. Due to the adverse effect on feed intake and lactation, it is worthwhile for the dairy farmer to carefully observe and adjust the size of the water trough, especially in summer.

The average water consumption of this study was 43 ± 14 m^3^/cow/yr in Hinchu County (Table 1). The national average water consumption of dairy cattle was 83.2 m^3^/cow/yr [32]. For the study, the dairy farms prepared sawdust or rice husk bedding as a buffer at the resting areas. The purpose of using the bedding was to absorb the urine or water from the cow dung. The farmer will use the scraper type manure cleaning system or the shovel truck to remove the wet and dirty bedding. This practice helps to comply with Taiwan’s environmental regulations. 

The ratio distributions of various water uses among the five dairy farms are shown in Figure 3. Farm# 4 was the only dairy farm that added some bio-agents (e.g., specific enzymes) into the water sprinkler system with timer control for reducing malodors in the farm and inhibiting insect proliferation. On Farm#3, to avoid any injuries resulting from the wet and slippery bedding, the bedding was shoveled and the pen was water cleaned once a day. The Farm#3 only applied array fans for cooling the cattle pens and seldom used a water sprinkler system, which might cause heat stress on cattle [33]. 

It is important to supply lactating cows with fresh, clean, and adequate water after milking. Otherwise, low DMI might result in low milk production efficiency and poor milk quality [31]. For cleaning and replenishing the water troughs, the volume of water use for Farms# 3 and #4 was only 1.6 and 1.9% of their total water consumption, respectively. The cleaning frequency of cleaning drinking water troughs on the Farm# 3 was very low, only twice every month. This might cause an increase in the number of microorganisms in the water trough and compromise water quality. Because of poor water quality, cows may reduce their water intake and indirectly affect their milk production, resulting in economic losses [34]. By comparison, Farm# 1 cleaned and replenished the water troughs at least once a day, but twice a day in summer. This may provide a guarantee for the maintenance of raw milk production (Table 1). Farm# 3 had the heaviest workload among all the dairy farms. On average, each employee (including the employer) needs to take care of 70 cows (Table 1), which may be one of the reasons why Farm#3 has no extra labor to keep the water troughs clean.

### 3.5. Impact Level Assessment 

The severity of water scarcity conditions, i.e., higher WSI, in some counties and cities of northern and central Taiwan was greater than in other counties or cities (Figure 4). The severity of water scarcity conditions in eastern and southern Taiwan were relatively low. The WSI of each county and city were closely related to local availability of water resources, water use, and precipitation changes. Based on the official statistical data, up to 92.9% of water in Taipei City was used for domestic use because of its greatest residence density in Taiwan [35]. Thus, the data of WSI in Taipei City were mostly affected by domestic water use. In contrast, the WSI in central Taiwan were heavily affected by agricultural water use. In particular, Changhua County and Yunlin County used about 95.8 and 83.6% of water for agriculture, respectively.

The stress-weighted WSF for raw milk production by the dairy farms in Hsinchu County was 9.4 H_2_O-eq/kg FPCM, which is lower than the studies in the Netherlands (33.4 H_2_Oeq/kg FPCM), Southeastern Australia (87.7 H_2_O-eq/kg FPCM), and China (11 H_2_O-eq/ kg FPCM) [8,9,12]. However, the data of stress-weighted WSF of this study was higher than the studies in Finland (3.8 H_2_Oeq/kg FPCM) and Ireland (0.4 H_2_Oeq/kg FPCM) [10,13]. 

The boundaries of the investigations, the inventory items, the time-scale dimensions of the data, the production patterns, and the technical and environmental factors were different for every study conducted by international research teams. For example, the study by De Boer et al. [9] assumed that each milking cow (including young stock) required 145 L of drinking and cleaning water per day. The study by Kuoppala et al. [36] calculated the daily drinking water of lactating cows according to their milk production. When a system boundary includes the stage of feed crop production, location of production, and the production type (irrigated versus rain-fed agriculture), the hotspots in the production chains and the magnitude of their impacts will greatly affect the impacts of milk production systems [13]. Thus, the comparability between studies will be limited.

### 3.6. Impact Mitigation Options for the Agri-Food Industry

The most important thing in WF assessment is how to use the information obtained from the assessment process to develop a water resource sustainable use strategy [37]. Due to the obvious temporal and spatial characteristics of Taiwan’s precipitation, and the uneven development of various regions, it is advisable to set up appropriate mechanisms in the future from the perspective of governmental management, i.e., by incorporating regional hydrology into one of the auditing requirements for the establishment or expansion of dairy farms. Another example would be guiding dairy farms to the eastern and southern parts of Taiwan through incentives or regulations, which will decrease the WSF but increase the WSP values. At the same time, it will help to slow down the increase of WSI in specific areas. However, the cost of transportation for raw milk and the availability of feeds such as concentrates may need to be overcome. 

A “groundwater fee policy” might be in the right direction of thinking, since almost all dairy farms use groundwater. Current groundwater users (e.g., dairy farmers) only pay for administrative application fees to the authority. The cost of infrastructure construction and electricity costs of motor operation are paid individually by the dairy farmers. A reasonable price mechanism should be established to optimize the groundwater utilization [38]. 

Raw material suppliers or manufacturers in the food sector are responsible to educate consumers who wish to be informed about the origin of their food and the sustainability of their production style [39,40,41]. From the perspective of dairy farms, any investments for promoting production efficiency, developing renewable water sources with low environmental impacts, and conserving the surface runoff from rainfall as water resource should be encouraged.

### 3.7. Limitations of This Study/Monthly Variation of the Water Stress Index (WSI)

Precipitation in Taiwan has spatial and seasonal changes, which can affect the use of water at different points in time. Thus, it might be more suitable for assessing the monthly WSI analysis for the counties and cities in Taiwan [42]. However, there is still no monthly data for water consumption and milk productivity for all dairy farms located in all counties and cities available. The data in Table 3 showed that different farm management styles can make various water consumption efficiencies. In addition, we cannot collect all milk productivity data from individual dairy farms. Thus, the relationship between the WSI data and the milk productivity of each dairy farm located in the 16 counties cannot be discussed in this study. Application of the method of Ridoutt and Pfister [4] might result in misjudgment of the relevant results. According to the sample size and sample representativeness of this study, samples will be collected from all 16 cities and counties in the next study. At least three management types of dairy farms are included for sample collection in each city or county. In the future, if accurate and clear spatial scale data could be obtained, it would help to improve the accuracy of the impact assessment. 

## 4. Conclusions

This is the first study in Taiwan that combines the production of raw milk with regional hydrological characteristics, which might lead to the improvement of existing regulations. The water stress in various regions of Taiwan varies considerably (WSI ranges from 0.05 to 1), which also represents the feasibility of reducing environmental impact by changing the industrial location. Additionally, nearly half of the forage is imported, resulting in higher raw milk production costs than in the USA and New Zealand. Thus, dairy farming should operate by means of more environmentally-friendly and sustainable ways in Taiwan according to the water footprint. In practice, altering the production location and promoting production efficiency of the domestic dairy industry will make it more feasible to reduce environmental impacts than by changing the source of dietary intake (although its key influence has been reported by many studies). Water is of absolute importance for the production and profitability of raw milk, so improving the WF requires precise implementation. In the future, more research and intensive information is needed to achieve better balance between raw milk production and environmental protection. Regional water stress is only one part of many environmental impacts and issues of concern, such as water pollution, eutrophication, and greenhouse effects.

## Figures and Tables

**Table 1:** Characteristics of dairy farm systems in the Hsinchu country of Taiwan from May 2018 to April 2019.

Parameters	Units	Farm Number					Average	Total
		1	2	3	4	5		
Livestock								
Number of heifers < 6 mths old	head	22	69	36	27	64	44	-
Number of heifers > 6 mths old	head	60	54	73	122	73	76	-
Number of bred heifer	head	23	48	19	30	56	35	-
Number of milkers	head	86	178	77	200	160	140	-
Number of dry cows	head	12	25	6	16	16	15	-
Total dairy cattle	head	203	374	211	395	369	310 ± 95	1552
Workload	heads/labor	41	47	70	56	53	53 ± 11	-
Raw milk fat content	%	3.81	3.87	3.86	3.74	3.94	3.85	-
Raw milk protein content	%	3.33	3.24	3.17	3.27	3.37	3.27	-
Raw milk production	t	919	1798	681	1713	1683	1359	-
FPCM ^a^	t	316	617	234	588	578	467	2333
Daily raw milk yield	kg	29.3	27.6	24.2	23.5	28.9	26.7	-
Daily FPCM yield	kg	10.1	9.5	8.3	8.0	10.0	9.2	-
Number of water meters	set	2	4	2	2	2		
Total water consumption	m^3^/yr	11,544	19,356	4176	16,812	15,576	13,493 ± 5923	67,464
Water consumption per head	m^3^/head/yr	56.1	51.8	19.9	43.0	43.4	43 ± 14	
95% of Water consumption ^b^	L/kg FPCM	34.9	29.9	17.0	27.3	25.7	27 ± 7	-
Feed								
Chinese Pennisetum	t	-	-	-	-	200	-	200
Pangola grass	t	406.5	-	72.5	-	-	-	479

^a^ FPCM: Fat and protein corrected milk (IDF, 2010). ^b^ 95% of water consumption is used as raw milk production (economic approach). Data are presented on a yearly basis for the on-farm area unless otherwise stated.

**Table 2:** Dairy farms’ water trough parameters.

Parameters	Units	Farm No.				
		1	2	3	4	5
Available length of the water trough	cm/head	14.0	10.1	3.7	5.9	13.4
Water trough supply	heads/water trough	16.9	31.2	26.5	43.9	21.7
Average water trough depth	cm	27.5	29.2	30.75	23.3	35
Average water trough width	cm	45	54.2	71.25	39.4	30

**Table 3:** Monthly amount and ratio of water consumption for the selected dairy farms.

Item No.	Categories of Water Consumption (m^3^)	Farm No.					Total (m^3^)/ Ratio (%)
		1	2	3	4	5	
	Water meter	962 ± 390 (100%)	1613 ± 186 (100%)	348 ± 65 (100%)	1401 ± 169 (100%)	1298 ± 473 (100%)	5622 (100%)
1	Water used in the milking room	146 ± 13 (15%)	146 ± 73 (9%)	85 ± 9 (24.4%)	150 ± 46 (11%)	155 ± 31 (12%)	552 (9.8%)
2	Clean and supplement the water trough	169 ± 68 (18%)	105 ± 18 (7%)	5.5 ± 0.8 (2%)	27 ± 6 (2%)	75 ± 29 (6%)	382 (6.8%)
3	Cooling water	108 ± 140 (11%)	301 ± 278 (19%)	6 ± 20 (2%)	102 ± 117 (7%)	239 ± 250 (18%)	756 (13.4%)
4	TMR mixed water	35 ± 3 (4%)	61 ± 0 (4%)	18 ± 2 (5.3%)	150 ± 8 (11%)	79 ± 37 (6%)	343 (6.1%)
5	Cleaning water	11 ± 13 (1%)	179 ± 61 (11%)	4.4±2.7 (1.3%)	151 ± 92 (11%)	35 ± 30 (3%)	380 (6.8%)
6	Miscellaneous work including disinfection	0.4 ± 1.4 (0%)	5.4 ± 1.4 (0.3%)	1.0 ± 0 (0.3%)	80 ± 31 (6%)	3.8 ± 0.6 (0.3%)	91 (0.2%)
7	Drinking meter	480 ± 175 (50%)	811 ± 169 (50%)	222 ± 47 (64%)	708 ± 93 (51%)	690 ± 209 (53%)	2911 (52%)
Estimated total (Item 1–7)		950	1608	342	1368	1277	5415

All farms use sawdust or rice husk as bedding. Recording time period: May 2018 to October 2018 and April 2019.

**Table 4:** The water stress index (WSI) in various counties and cities in Taiwan.

Regions	County/City	Water Stress Index (WSI)	Stress-Weighted Water Scarcity Footprint (WSF)	Water Scarcity Productivity (WSP)
			H_2_O_eq_/kg FPCM	Fat and Protein Corrected Milk (FPCM)-kg/m^3^-Water
Northern Taiwan	New Taipei City (NTPC)	0.73	32.7	0.051
	Taipei City (TPE)	1	44.8	0.037
	Taoyuan City (TYN)	1	44.8	0.037
	Hsinchu Country (HSZ)	0.21	9.4	0.178
	Ilan Country (ILA)	0.08	3.6	0.468
Central Taiwan	Miaoli Country (ZMI)	0.1	4.5	0.375
	Taichung Country (TXG)	1	44.8	0.037
	Changhua Country (CHW)	1	44.8	0.037
	Nantou Country (NTC)	0.05	2.2	0.749
	Yunlin Country (YUN)	0.99	44.3	0.038
Southern Taiwan	Chiayi Country (CYI)	0.05	2.2	0.749
	Tainan City (TNN)	0.77	34.5	0.049
	Kaohsiung City (KHH)	0.14	6.3	0.268
	Pingtung Country (PIF)	0.12	5.4	0.312
Eastern Taiwan	Taitung Country (TTT)	0.15	6.7	0.250
	Hualien Country (HUN)	0.09	4.0	0.416

Keelung City, Hsinchu City and Chiayi City excluded the assessment due to lack of water use information.

**Table 5:** Acceptance and pricing rule for the acquisition of raw milk by dairy processing plants in Taiwan.

Solids-Not-Fat (%)		8.00–8.16			8.17–8.32			8.33–8.48			8.49–8.64			8.65–8.80			8.81–8.99			9.00–9.17			Above 9.18		
	Seasons	C	T	W	C	T	W	C	T	W	C	T	W	C	T	W	C	T	W	C	T	W	C	T	W
Milk Fat (%)																									
2.8		0.54	0.70	0.77	0.55	0.71	0.77	0.55	0.71	0.78	0.55	0.72	0.78	0.56	0.72	0.79	0.56	0.72	0.79	0.56	0.73	0.79	0.57	0.73	0.80
2.9		0.60	0.77	0.84	0.61	0.77	0.84	0.61	0.78	0.85	0.61	0.78	0.85	0.62	0.79	0.85	0.62	0.79	0.86	0.62	0.79	0.86	0.63	0.80	0.86
3.0		0.66	0.84	0.90	0.67	0.84	0.91	0.67	0.85	0.91	0.67	0.85	0.92	0.68	0.85	0.92	0.68	0.86	0.92	0.68	0.86	0.93	0.69	0.86	0.93
3.1		0.67	0.85	0.92	0.68	0.85	0.92	0.68	0.86	0.93	0.68	0.86	0.93	0.69	0.87	0.93	0.69	0.87	0.94	0.69	0.87	0.94	0.70	0.88	0.94
3.2		0.70	0.88	0.94	0.70	0.88	0.95	0.70	0.89	0.95	0.71	0.89	0.96	0.71	0.89	0.96	0.71	0.90	0.96	0.72	0.90	0.97	0.72	0.90	0.97
3.3		0.71	0.89	0.95	0.71	0.89	0.96	0.72	0.90	0.97	0.72	0.90	0.97	0.73	0.91	0.98	0.73	0.92	0.98	0.74	0.92	0.99	0.74	0.93	0.99
3.4		0.71	0.90	0.96	0.72	0.90	0.97	0.73	0.91	0.98	0.74	0.92	0.99	0.74	0.93	0.99	0.75	0.93	1.00	0.76	0.94	1.01	0.76	0.95	1.01
3.5		0.72	0.90	0.97	0.73	0.91	0.98	0.73	0.92	0.98	0.74	0.93	0.99	0.75	0.93	1.00	0.75	0.94	1.01	0.76	0.95	1.01	0.77	0.95	1.02
3.6		0.73	0.91	0.98	0.73	0.92	0.98	0.74	0.92	0.99	0.75	0.93	1.00	0.75	0.94	1.01	0.76	0.95	1.01	0.77	0.95	1.02	0.77	0.96	1.03
3.7		0.73	0.92	0.98	0.74	0.92	0.99	0.75	0.93	1.00	0.75	0.94	1.00	0.76	0.94	1.01	0.77	0.95	1.02	0.77	0.96	1.03	0.78	0.97	1.03
3.8		0.74	0.92	0.99	0.75	0.93	1.00	0.75	0.94	1.00	0.76	0.94	1.01	0.77	0.95	1.02	0.77	0.96	1.03	0.78	0.97	1.03	0.79	0.97	1.04
3.9		0.74	0.93	1.00	0.75	0.94	1.00	0.76	0.94	1.01	0.77	0.95	1.02	0.77	0.96	1.02	0.78	0.96	1.03	0.79	0.97	1.04	0.79	0.98	1.05
4.0		0.75	0.93	1.00	0.76	0.94	1.01	0.76	0.95	1.02	0.77	0.96	1.02	0.78	0.96	1.03	0.78	0.97	1.04	0.79	0.98	1.04	0.80	0.99	1.05

(Unit: USD/kg raw milk). 1. C: Cool season; T: Temperate season; W: Warm season. The cool season is from December to March; the temperate season is April, May, October, and November; and the warm season is from June to September. 2. If the milk fat percentage is less than 2.8% or the solids-not-fat (SNF) percentage is less than 8.0%, the price is not counted. 3. The price per kilogram of raw milk is based on the list.
